# The Decline in Intrinsic Connectivity Between the Salience Network and Locus Coeruleus in Older Adults: Implications for Distractibility

**DOI:** 10.3389/fnagi.2020.00002

**Published:** 2020-01-31

**Authors:** Tae-Ho Lee, Sun Hyung Kim, Benjamin Katz, Mara Mather

**Affiliations:** ^1^Department of Psychology, Virginia Tech, Blacksburg, VA, United States; ^2^Department of Psychiatry, The University of North Carolina at Chapel Hill, Chapel Hill, NC, United States; ^3^Department of Human Development and Family Science, Virginia Tech, Blacksburg, VA, United States; ^4^Davis School of Gerontology, University of Southern California, Los Angeles, CA, United States

**Keywords:** functional connectivity, older adults, locus coeruleus, fMRI, resting-state activity

## Abstract

We examined functional connectivity between the locus coeruleus (LC) and the salience network in healthy young and older adults to investigate why people become more prone to distraction with age. Recent findings suggest that the LC plays an important role in focusing processing on salient or goal-relevant information from multiple incoming sensory inputs (Mather et al., 2016). We hypothesized that the connection between LC and the salience network declines in older adults, and therefore the salience network fails to appropriately filter out irrelevant sensory signals. To examine this possibility, we used resting-state-like fMRI data, in which all task-related activities were regressed out (Fair et al., 2007; Elliott et al., 2019) and performed a functional connectivity analysis based on the time-course of LC activity. Older adults showed reduced functional connectivity between the LC and salience network compared with younger adults. Additionally, the salience network was relatively more coupled with the frontoparietal network than the default-mode network in older adults compared with younger adults, even though all task-related activities were regressed out. Together, these findings suggest that reduced interactions between LC and the salience network impairs the ability to prioritize the importance of incoming events, and in turn, the salience network fails to initiate network switching (e.g., Menon and Uddin, 2010; Uddin, 2015) that would promote further attentional processing. A chronic lack of functional connection between LC and salience network may limit older adults’ attentional and executive control resources.

## Introduction

Studies have suggested that attentional control deficits in older adults are due to age-related changes of the frontal system, particularly the frontoparietal network, composed of the dorsolateral prefrontal cortex (DLPFC), middle frontal gyrus (MFG), inferior frontal gyrus (IFG), and anterior/inferior parietal lobule (Gazzaley et al., 2005; Campbell et al., 2012; Kennedy and Mather, in press), such that older adults typically show reduced activation in the frontoparietal network compared with younger adults (Ferreira and Busatto, 2013; Geerligs et al., 2014; Grady et al., 2016). As a result, older adults become more prone to distraction (Gazzaley et al., 2008; Schmitz et al., 2010, 2014; Lee et al., 2018) and this increased distractibility is linked to the general cognitive and emotional declines observed in older adults (Hasher and Zacks, 1988; Verhaeghen and Cerella, 2002; Whalley et al., 2004; Yerys et al., 2009; Wadlinger and Isaacowitz, 2011; Kennedy and Mather, in press).

The critical role of the frontoparietal network in the maintenance of attentional focus and execution (Corbetta and Shulman, 2002; Petersen and Posner, 2012; Scolari et al., 2015) and its age-related declines (Gazzaley et al., 2008; Schmitz et al., 2010, 2014; Lee et al., 2018) has been investigated extensively by examining frontoparietal network activity. However, examining the frontoparietal network in isolation may miss important aspects of age differences in the functional circuitry of attention, as many human imaging studies and theoretical models have posited that attentional processes require large-scale brain-systems-level interactions between intrinsic neural networks (Menon and Uddin, 2010; Uddin, 2015). That is, attentional processing does not result from the activity of one single network alone. In particular, neuroimaging research suggests that the default mode network, including the medial PFC (MPFC), posterior cingulate cortex (PCC), and precuneus, the salience network, which includes the anterior cingulate cortex and anterior insula, and the frontoparietal network each are involved in implementing attentional processes in the brain, and also that they interact with each other. Converging evidence indicates that the salience network helps initiate frontoparietal network control of attentional processing for prioritized input (Seeley et al., 2007; Goulden et al., 2014). Thus, from a theoretical perspective, when the salience network does not filter out incoming sensory inputs appropriately and continues to activate the frontoparietal network for every sensory signal, it may lead to unnecessary depletion of limited neural resources and impairment of focused, goal-directed attentional processes. Consistent with this possibility, deficits in the salience network (e.g., decreased neural activation and connectivity between local regions within the network) are associated with attention deficits in both younger and aging adults (Lopez-Larson et al., 2012; Song et al., 2014; Cai et al., 2015; Zhao et al., 2017).

However, the underlying neural mechanisms involved in attention deficits in the brain are still largely unclear. As discussed above, some evidence suggests that attention deficits are due to a failure of the salience network in prioritizing incoming sensory inputs at the initial stage (Lopez-Larson et al., 2012; Song et al., 2014; Cai et al., 2015; Zhao et al., 2017), whereas other evidence indicates that impaired processing efficiency within the frontoparietal network *per se* reduces the maintenance of attentional focus and execution (Zhou et al., 2011; Campbell et al., 2012; Li et al., 2013). That is, it is not clear whether increased age-related distractibility is due to a failure of ignoring distractors (i.e., salience errors) or maintaining concentration on a task (i.e., execution errors).

In this context, we note that several studies have highlighted the role of the locus coeruleus (LC), a small nucleus in the brainstem that constitutes the major source of norepinephrine (NE), in determining processing selectivity in the brain. LC releases NE to almost the entire brain throughout its widespread efferent projections, namely the LC-NE system (Foote et al., 1983; Aston-Jones and Cohen, 2005; Bouret and Sara, 2005; Sara, 2009). The neural activity patterns of the LC-NE system, between tonic and phasic responses, determine changes in large-scale neural network configurations (Zerbi et al., 2019) to bias neural processing selectively towards goal-relevant events (Mather and Harley, 2016; Mather et al., 2016). Recent fMRI studies have demonstrated that attentional selectivity tends to be less pronounced when the LC responds to external events indiscriminately (Lee et al., 2014; Clewett et al., 2018). This reduced specificity of LC responses may induce the loss of attentional focus in older adults, regardless of the saliency level of incoming sensory signals (Lee et al., 2018).

Neurobiological studies indicate that the salience network provides input to the LC-NE system (Ullsperger et al., 2010; Unsworth and Robison, 2017; Zerbi et al., 2019), suggesting that communication between the salience network and LC-NE system is involved in prioritizing sensory events. Neurobiological and theoretical models support this view by showing that the LC-NE system has a reciprocal connection with the salience network, which in turn may increase the selectivity of attentional processes shaped by the salience network (Jodo et al., 1998; Rajkowski et al., 2000; Markovic et al., 2014; Mather and Harley, 2016). Consistent with this influence of the LC, the salience network shows decreased activity when LC-NE system activity is attenuated by the *β*-adrenoreceptor blockade (Hermans et al., 2011). Given the involvement of the LC in salience network processes, it is possible that increases in age-related distractibility are partially due to diminished neural connectivity between the salience network and the LC-NE system, which in turn means the salience network cannot prioritize important information from among multiple events to appropriately activate the frontoparietal network. However, to date, the potential connection between the LC-NE system and salience networks that may be linked to increased attentional distractibility in older adults has not been fully investigated.

In the current study, we examined age-related differences between LC and salience network connectivity. We hypothesized that the functional connections between the LC and the salience network decline in older adults (hypothesis 1). As a result, even in the absence of task demands, the salience network continuously and indiscriminately switches from the task-negative network to the frontoparietal network (Menon and Uddin, 2010; Uddin, 2015; hypothesis 2). To test these possibilities, we focused on the functional connectivity between the LC and salience network as a function of age group (younger vs. older adults) by using a pre-existing fMRI dataset (Lee et al., 2018). Given that this public data is task-based, we adopted a resting-state-like fMRI approach, in which all task-related activities were regressed out. Thus, our analyses can be more focused on the native and intrinsic connectivity between regions, without the influence of task-related signal fluctuations (Fair et al., 2007; Elliott et al., 2019).

## Procedure and Methods

### Open fMRI Dataset and Preprocessing

The dataset used in the current study, from the open-fMRI repository^1^, consists of 28 healthy younger adults (YA: M_age_ = 24.39 years, age range = 18–34; nine females) and 24 healthy older adults (OA: M_age_ = 66.95 years, age range = 55–75; nine females). There were no significant differences between groups in terms of intellectual ability, as measured by either education or the Wechsler Test of Adult Reading (Wechsler, 1981): *M*_education_: YA = 16.85 vs. OA = 16.38 years; *M*_Wechsler Test of Adult Reading_: YA = 43.96/50 vs. OA = 39.75/50.

We used high-resolution structural images for our analyses (MPRAGE; TR = 1,950 ms; TE = 2.26 ms; FA = 7°; 1-mm isotropic voxel; FOV = 256 mm) and five identical task runs of the dataset (142 volumes for each EPI; 41 interleaved 4-mm slices with no gap; TR = 2,000 ms; TE = 25 ms; FA = 90°; matrix size = 64 × 64; FOV = 256), in which participants were exposed to different levels of auditory and visual stimuli in terms of emotional arousal and saliency (Lee et al., 2018). The images were acquired on a Siemens 3T Magnetom Trio with a 32-channel matrix. Preprocessing was performed using the FMRIB software library (FSL^2^) including skull stripping and tissue mask segmentation (CSF/WM/GM) of structural images after bias-field correction; first three volumes cut, motion correction, smoothing using a Gaussian kernel of FWHM 6 mm slice-timing correction, grand-mean intensity normalization of the entire 4D data set, ICA denoising using ICA-AROMA^3^, and 0.001–0.08 Hz linear-trends filtering and regressing out CSF/WM signal. To examine intrinsic functional connectivity unrelated to task-induced activity, all task-related activities were also regressed out (Fair et al., 2007; Elliott et al., 2019). For details of the task-activity related connectivity analysis and the task itself, please see the previous work (Lee et al., 2018). Finally, the preprocessed resting-state-like fMRI data were transformed to standard MNI 2-mm brain through the non-linear transformation matrix of structure-to-standard-brain using the Advanced Normalization Tool library (ANTs^4^).

Through the preprocessing, we identified that one of the OA participant’s data was contaminated by severe motion across runs (mean framewise displacement, FD, and DVAR were 0.57 mm and 63.36, respectively). Because the severe motion can significantly reduce the estimation reliability of the functional connectivity (Power et al., 2012), we excluded this participant’s data from further analysis (final data *N* = 51; mean FD and DVAR were 0.054 mm and 12.45, respectively).

### Whole-Brain Functional Connectivity Analysis With LC Seed

Based on our hypothesis, we focused on the functional connectivity between LC and salience networks and ran a whole-brain functional connectivity analysis. To this end, we first extracted the mean time-series of LC activity from the preprocessed and task-activity-free fMRI data using a standard structural LC mask (Edlow et al., 2012). Using this LC time course, a multiple regression analysis was performed to estimate individual lower-level functional connectivity maps (i.e., seed correlation map) for each fMRI run using FSL FEAT. Then, a second-level fixed-effects analysis was performed across each participant’s functional runs. Finally, individual-level LC seed connectivity maps were inputted into group-level analysis of mixed effects using cluster correction (FLAME 1 + 2; *Z* > 2.57; *corrected* *P* = 0.05) with two between-group contrasts (YA > OA) and (YA < OA).

### Connectivity Network ROI Analysis With Salience Network Seed

**Figure 1:**
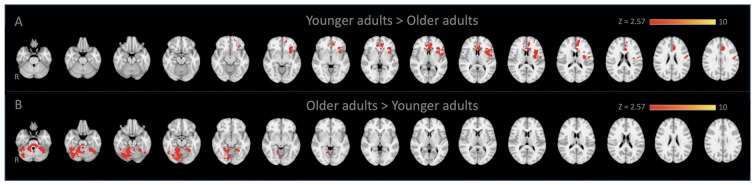
Locus coeruleus (LC)-seed based whole-brain functional connectivity results. Voxels that showed stronger connectivity with the LC for **(A)** younger adults than older adults (spatial cross-correlation of 0.345 with predetermined salience network maps: Smith et al., 2009; Laird et al., 2011) and **(B)** vice versa.

An additional network-ROI analysis was conducted to examine how the salience network may be coupled with the frontoparietal network natively beyond the influence of task-related activities. To this end, from the group-level LC seed-based analysis (see Figure 1A), we selected significant voxels showing age group differences within the previously identified meta-analysis salience network template (intrinsic network mask #4 in Laird et al., 2011), and extracted mean time course of activity of those voxels (salience network seed). The aforementioned connectivity estimation procedure was repeated for each individual and we extracted connectivity parameter estimates from the frontoparietal and default-mode masks (Smith et al., 2009; Laird et al., 2011) as an index of the salience connectivity strength between these two networks.

## Results

Whole-Brain connectivity analysis of the LC between age groups showed that, compared to YA, OA exhibited significantly reduced functional connectivity of the LC with the anterior cingulate cortex, anterior insula, and putamen (Figure 1A). These regions are considered to be the core of the salience network (Menon and Uddin, 2010; Uddin, 2015). To confirm that these regions showed reduced functional connectivity in OA constitute the salience network, additional spatial cross-correlation analysis with the previously defined salience network template (i.e., intrinsic network mask #4 in Laird et al., 2011) was performed and it showed a high degree of similarity (*r* > 0.358). This result indicates that the LC is connected to the salience network more strongly in YA than OA, suggesting that the salience network activity is coupled strongly with LC activity in YA but not in OA (Figure 1A; Table 1A). Also, OA showed more connectivity of the LC with initial visual processing regions, including the fusiform gyrus extended from occipital to temporal regions, the lingual gyrus and the inferior temporal gyrus (Figure 1B; Table 1B).

**Table 1:** Brain regions within significant clusters on the LC seed-based whole-brain connectivity analysis between younger adults (YA) and older adults (OA).

			MNI coordinates		
	H	Z	*x*	*y*	*z*
**A. YA > OA**					
Cluster 1 (*k* = 1,398)	
Central opercular cortex	L	3.64	−42	4	6
Putamen	L	3.63	−28	−2	12
Parietal operculum	L	3.50	−34	16	10
Insular cortex	L	3.16	−34	16	−8
Inferior frontal gyrus	L	2.73	−54	16	6
Frontal orbital cortex	L	2.71	−34	24	−8
Frontal pole	L	2.71	−18	56	−16
Cluster 2 (*k* = 1198)		
ACC		3.23	−6	40	4
Paracingulate cortex		3.10	−8	46	14
**B. OA > YA**						
Cluster 1 (*k* = 4,167)	
Lingual gyrus	R	3.59	14	−56	−8
Temporal occipital fusiform cortex	R	3.52	42	−48	−22
Occipital fusiform gyrus	R	3.37	32	−70	−16
Temporal occipital fusiform cortex	L	3.18	−28	−52	−18
Lingual gyrus	L	2.77	−18	−46	−12
Inferior temporal gyrus	R	2.73	50	−46	−22
Temporal fusiform cortex	L	2.63	−30	−40	−22
posterior division

*Please note the reported region labels are the peak value locations within the Harvard-Oxford structure atlas on each cluster (H = hemisphere; Z = z-value)*.

**Figure 2:**
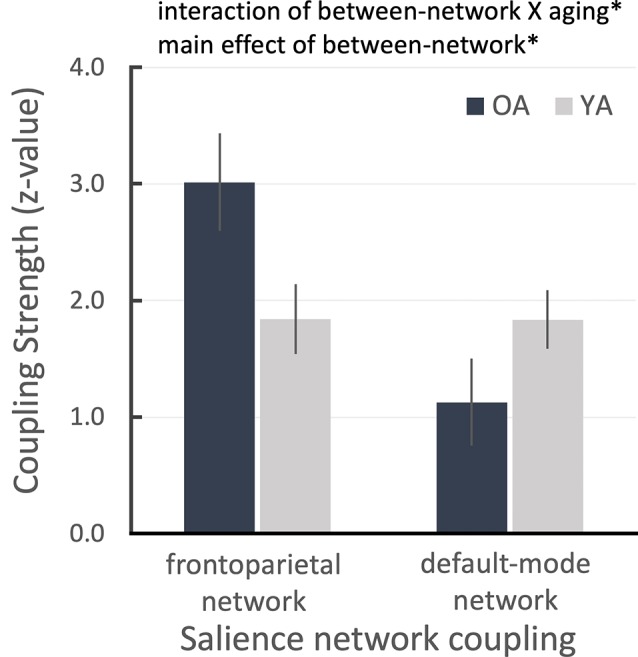
ROI results for salience network seed-based functional connectivity analysis showing the salience network couplings with frontoparietal and default networks as a function of aging group. Error bars denote the standard error term. *P < 0.05.

Due to the reduced LC and salience network connectivity in OA, we hypothesized that the OA’s salience network could not differentiate between important and unimportant sensory inputs and that it might continuously switch the network configuration from the default-mode network to the frontoparietal network based on the previous model of how the salience network coordinates network switching (Menon and Uddin, 2010; Uddin, 2015). To test this hypothesis, we extracted connectivity parameter estimates from the previously defined frontoparietal and default-mode masks (Smith et al., 2009; Laird et al., 2011) as an index of the salience connectivity strength between these two networks, from the salience network seed-based connectivity maps for each age group (see “Connectivity Network-ROI Analysis With Salience Network Seed” section). We found that the salience network was more coupled with the frontoparietal network in OA than YA, even though all task-related activities were initially regressed out. Conversely, OA’s salience network was less coupled with the default-mode network than in YA participants. This pattern was confirmed by a between-network condition (2: salience-frontoparietal, salience-default) and aging group (2: OA, YA) repeated-measures ANOVA, which revealed a significant cross-over interaction, *F*_(1,49)_ = 6.135, *p* = 0.017, partial-$\eta_{\text{p}}^{2}$ = 0.111, and a main effect of between-network condition, *F*_(1,49)_ = 6.215, *p* = 0.016, partial $\eta_{\text{p}}^{2}$ = 0.113 (Figure 2). That is, despite all task-related activities having been regressed out before the connectivity analyses, the OA’s salience network is still excessively coupled with the task-positive network compared to YA and the task-negative network.

**Figure 3:**
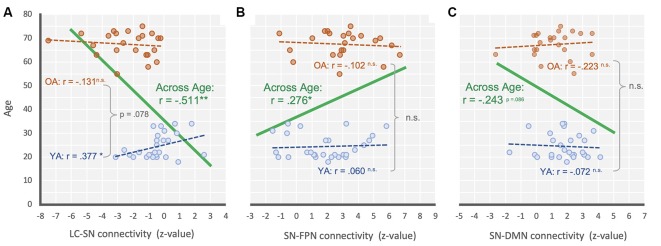
Scatter plot illustrating the relationship between **(A)** LC-SN, **(B)** SN-FPN, and **(C)** SN-DMN connectivity and across age for across range (green), YA only (blue) and OA only (orange). **P < 0.001; *P < 0.05; n.s., non-significant.

Additional correlation analyses were performed to see the relationship between age and each connectivity strength (i.e., LC-SN, SN-DMN, SN-FPN). As a result, we found that there was a significant negative relationship between the LC-SN connectivity strength and age overall, *r* = −0.511, *p* < 0.001, suggesting that the LC-SN connectivity decreased with age (Figure 3A). For the groups separately, YA’ age showed a significant positive correlation with the connectivity strength, *r* = 0.377, *p* < 0.05, but OA did not show such relationship, *r* = −0.131, *p* = 0.550. We additionally compared the correlation coefficients between age groups, and found there was a marginally significant difference, *z* = −1.76, *p* = 0.078. In the SN-FPN, there was a significant relationship between the connectivity strength and age overall, *r* = 0.276, *p* = 0.05, indicating that the increased SN-FPN connectivity, even in the absence of attentional demands (i.e., resting state), was associated with age (Figure 3B). On their own, neither YA and OA showed significant relationships between age and connectivity strength, *r*s < ± 0.09, *p*s > 0.662, and no difference between correlation coefficients between groups, *z* = −0.52, *p* = 0.603. In the SN-DMN, there was a marginally significant relationship between the age and connectivity strength, *r* = −0.243, *p* = 0.086, showing that there is a linear trend toward a decrease in the SN-DMN connection with age (Figure 3C). For each group separately, neither YA and OA showed significant relationships between age and connectivity strength, *r*s < ± 0.11, *p*s > 0.635, and no difference between group coefficients, *z* = 0.59, *p* = 0.552. In sum, these correlational results were consistent with the main findings that showed age-related mean differences of connectivity between intrinsic networks and regions.

## Discussion

It is impossible to process every stimulus around us simultaneously at any given moment. Thus, we need to select what is important from our surroundings, and focus mental resources on those selected for sufficient periods of time to be able to process them (Treisman, 1960; Carrasco, 2011; Wolfe and Horowitz, 2017). However, this attentional control ability declines with age, and older adults appear more susceptible to distraction and interference compared to their younger counterparts. These declines have been demonstrated through an extensive body of behavioral research (Madden et al., 1994; West and Alain, 2000; Verhaeghen and Cerella, 2002; Healey et al., 2008); subsequent imaging work has established decreases in the responsiveness of neural attentional control resources in older adults as well (Milham et al., 2002). Eventually, this increased distractibility in older adults is associated with more general cognitive and emotional declines, although perspectives differ on the fundamental nature of these issues (Hasher and Zacks, 1988; Verhaeghen and Cerella, 2002; Whalley et al., 2004; Yerys et al., 2009; Wadlinger and Isaacowitz, 2011; Kennedy and Mather, in press). Some of this work includes convergent evidence from working memory research that specifically explores the effects of distraction in older adults (e.g., Chadick et al., 2014); we note that one recent review article proposes an LC-NE specific account of differences in performance in both working memory and attentional control (Unsworth and Robison, 2017). However, despite work that confirms both the behavioral and neural implications of declines in attentional control, our understanding of the fundamental underlying neural mechanisms of how and why older adults are prone to distraction remains somewhat incomplete. Dysregulation in LC-NE function remains one compelling, but largely unexplored, explanation for non-optimal frontoparietal network activity during aging.

In the present study, we examined age-related functional connectivity differences based on LC activity to provide insight into attentional deficits in older adults. In particular, we used a pre-existing task-based fMRI dataset that has exhibited task-based age deficits in attentional processing. Given the original findings from the dataset (Lee et al., 2018), we wanted to examine the intrinsic brain activity beyond task-based signal changes. A growing body of evidence suggests that the intrinsic brain activity (i.e., background neural signal fluctuation) plays a primary role in shaping task-based brain activity (Cole et al., 2014, 2016; Bzdok et al., 2016; Tavor et al., 2016). Using the intrinsic network extracted from the task-based fMRI is a useful approach to investigate the neural basis of task-evoked activity (Arfanakis et al., 2000; Fair et al., 2007; Elliott et al., 2019).

We found that older adults showed relatively reduced functional connectivity between the LC and the salience network compared with younger adults such that the signal fluctuation of the LC is coupled to the signal fluctuation of the salience network less in older adults than in younger adults. Instead, the LC showed stronger connectivity with visual processing regions, and the salience network was more coupled with the frontoparietal network compared to the default mode network, in older adults than younger adults, even though all task-related activities were regressed out. Together, these findings suggest that the interaction between LC the salience network fails to prioritize the importance of incoming events due to reduced functional connectivity, and in turn, the frontoparietal network is recruited continuously (e.g., Menon and Uddin, 2010; Uddin, 2015) even when it is unnecessary. This unnecessary coupling with the frontoparietal network, without differential priority for incoming events, may be the main source that limits older adults’ resources, inducing difficulty with sustaining attention and focusing on a specific task.

If the salience network helps to switch network configurations as suggested previously (Menon and Uddin, 2010; Uddin, 2015), the reduced connectivity observed here should contribute to older adults’ distractibility. Due to this reduced connectivity, the salience network may not get adequate feedback from the LC in responding to sensory inputs, and thus the older adults’ salience network may fail to ignore incoming, but non-important, events. This presupposes that the salience network is more likely to be influenced by the LC in computing saliency level, and biasing processing selectivity of the frontoparietal network afterward. Alternatively, the LC-NE system may achieve processing specificity from inputs of the salience network, and it directly regulates the attentional control system (Unsworth and Robison, 2017). If that is indeed the case, the increased distractibility observed in older adults is more likely to be an issue of inputs from the salience network to the LC-NE system, wherein the LC may not receive priority information from the salience network, leading to the LC sending excitatory inputs indiscriminately to the entire brain, including the frontoparietal network through its efferent projection (Corbetta and Shulman, 2002; Cohen et al., 2004). Unfortunately, given the correlational nature of functional connectivity analyses in human brain imaging (Joel et al., 2011), it is difficult to rule out other possible nodes through which communication between the LC and salience network activates the frontoparietal network. In any case, however, the current results suggest the importance of functional connectivity between the salience network and LC-NE system in a cascade of a focused attentional processes: the failure of functional communication between them unnecessarily depletes neural resources by increasing excessive processes even for task-incidental sensory information (Figure 1B) and inappropriate recruitment of the frontoparietal network (Figure 3).

Our findings are consistent with recent work that has established older-adults’ vulnerability to distraction, showing that age-related attentional deficits are more likely to be from a failure in the early selection of important stimuli rather than an inefficient process of execution at the later stage (Schmitz et al., 2010, 2014). Similarly, our previous findings demonstrated that older adults’ perception *per se* did not differ from that of younger adults in that older adults showed similar levels of perceptual specificity for task stimulus types (e.g., object and house) with younger adults, but older adults were worse at inhibiting irrelevant information (Lee et al., 2018). That is, older adults have fine perceptual representations of whatever is the focus of their attention, much as younger adults, but fail to inhibit representations that should be ignored. Extending previous findings, the current study provides a possible underlying mechanism suggesting that reduced functional communication between the LC and salience network in the brain contributes to age-related attention deficits. Thus, combining neurobiological studies that can isolate long-range neural connectivity between the LC and salience network more specifically (e.g., Kim et al., 2015) will be an important future direction to better understand these brain connectivity patterns and how they may be linked to age-related attentional deficits.

Finally, it is important to note that there are several limitations to our study. First, the LC is an exceptionally small structure in the brainstem, and thus it is hard to locate its location in an individual brain. Although we used the standard LC structure mask, given the low-resolution nature of EPI and the current 6-mm smoothing and MNI spatial normalization, the LC signal we extracted as a seed activity inherently includes other signals from neighboring regions within the brainstem such as periaqueductal gray (PAG) or ventral tegmental area (VTA). Therefore, in future work, it would be beneficial to utilize a neuromelanin sequence to locate individual LC location on the native space (e.g., Clewett et al., 2018) and extract a seed signal without smoothing (e.g., Alakörkkö et al., 2017). Second, the LC is often confounded by physio artifacts such as cardiac pulsation. In the current study, we applied the ICA-denoising process to correct physio noise in the brainstem. Although the ICA-denoising is a promising approach to mitigate physiological influence at the global level (Lee et al., 2014, 2018; Clewett et al., 2018), measuring individual respiration and cardiac pulse during future scans will be helpful to estimate the LC signal, as individual-based physio noise correction can be more focal and direct to the brainstem signal fluctuation correction (Glover et al., 2000).

## Data Availability Statement

The raw dataset can be found here: https://openneuro.org/datasets/ds001242. The dataset results from this study are available on request to the corresponding author.

## Ethics Statement

The dataset and code used in this study is from a public data repository. The original data collection procedure involving human participants was reviewed and approved by IRB at the University of Southern California.

## Author Contributions

All the authors contributed to the preparation of the manuscript. T-HL designed the study. Data were analyzed by T-HL with SK, BK, and MM.

## Conflict of Interest

The authors declare that the research was conducted in the absence of any commercial or financial relationships that could be construed as a potential conflict of interest.

## Footnotes

^1^https://openneuro.org/datasets/ds001242

^2^https://www.fmrib.ox.ac.uk/fsl

^3^https://github.com/maartenmennes/ICA-AROMA

^4^https://sourceforge.net/projects/advants
